# P-862. Impact of a Cefazolin for All Campaign on Perioperative Antibiotic Prophylaxis in Patients with a Penicillin Allergy Label

**DOI:** 10.1093/ofid/ofaf695.1070

**Published:** 2026-01-11

**Authors:** Katherine C Shihadeh, Tran Nguyen, Margaret M Cooper, Timothy C Jenkins

**Affiliations:** Denver Health, Denver, CO; Denver Health, Denver, CO; Denver Health, Denver, CO; Denver Health, Denver, CO

## Abstract

**Background:**

Cefazolin is the preferred antibiotic for prophylaxis of most surgical procedures. Despite its safety in patients with a penicillin allergy label (PAL), suboptimal antibiotics are frequently administered. To increase use of cefazolin as prophylaxis in patients with PAL, a Cefazolin for All campaign was implemented at Denver Heath in January 2024 and expanded to Obstetrics and Gynecology in June 2024. The purpose of this study was to evaluate the impact of the campaign on prophylactic cefazolin in patients with PAL.

**Figure:**
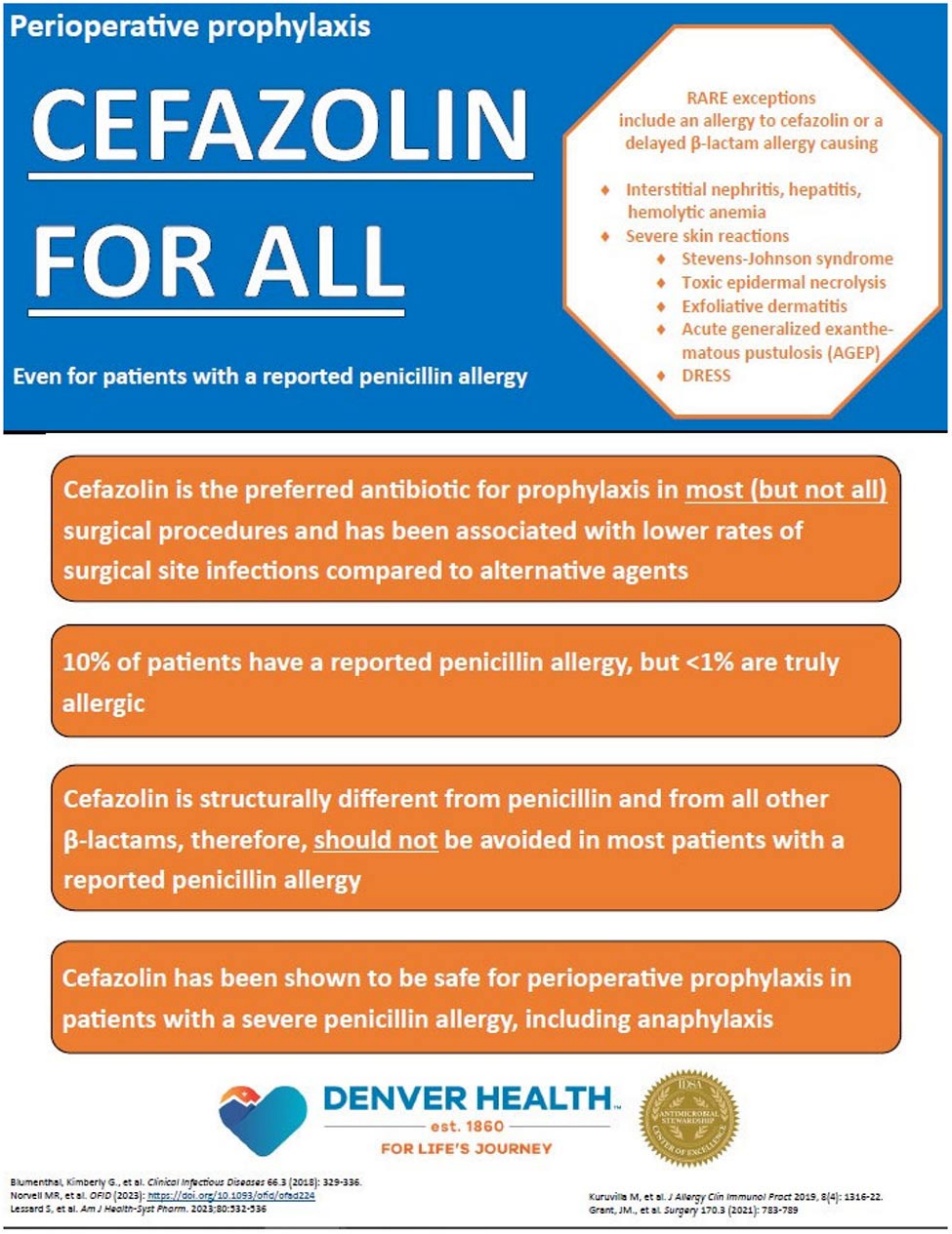

**Figure:**
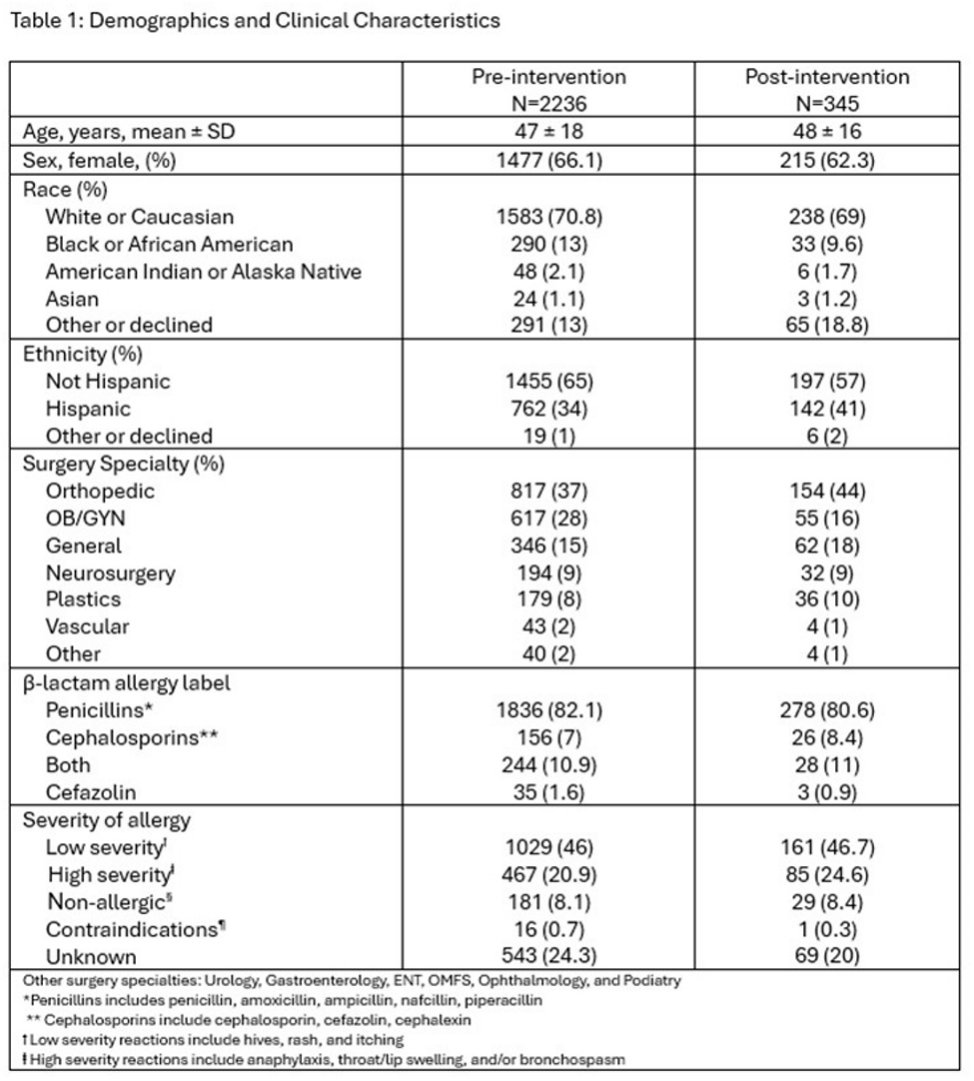

**Methods:**

This quasi-experimental study included adult patients who underwent an NHSN-reported procedure. Procedures for which cefazolin is not the recommended antibiotic were excluded. The pre-intervention period was 1/1/2017 – 12/31/2023 except OB/GYN procedures were from 1/1/2017 – 5/31/2024. The post-intervention group was 1/1/2024 – 1/31/2025 except OB/GYN procedures were from 6/1/2024 – 1/31/2025. The Cefazolin for All campaign included in-person education to surgeons and anesthesiologists, educational flyers posted in surgery clinics and perioperative areas (Figure 1), and updated institutional perioperative antibiotic guidance. The primary outcome was the proportion of patients with PAL who received guideline-concordant perioperative prophylaxis before and after the intervention. Secondary outcomes included occurrence of surgical site infection, readmission rate within 30 days, and the administration of diphenhydramine within 24 hours of surgery.

**Figure:**
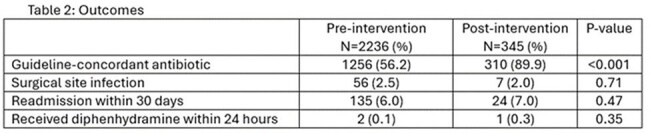

**Results:**

Of 21,507 patients in the pre-intervention group and 3,308 in the post-intervention group, 10.4% had PAL in each group. In the pre-intervention group, 56.2% received guideline concordant prophylaxis compared to 89.9% in the post-intervention group. A surgical site infection occurred in 2.5% of patients and 2.0% of patients in the pre- and post-intervention groups, respectively. There were 6.0% of patients and 7.0% of patients in the pre- and post-intervention groups readmitted within 30 days, respectively. Just 3 patients received diphenhydramine, 2 in the pre-intervention group and 1 in the post-intervention group.

**Conclusion:**

A Cefazolin for All campaign to increase cefazolin use for perioperative prophylaxis in patients with PAL was widely adopted without increased evidence of harm.

**Disclosures:**

All Authors: No reported disclosures

